# CRISPR-Based Approaches for Gene Regulation in Non-Model Bacteria

**DOI:** 10.3389/fgeed.2022.892304

**Published:** 2022-06-23

**Authors:** Stephanie N. Call, Lauren B. Andrews

**Affiliations:** ^1^ Department of Chemical Engineering, University of Massachusetts Amherst, Amherst, MA, United States; ^2^ Biotechnology Training Program, University of Massachusetts Amherst, Amherst, MA, United States; ^3^ Molecular and Cellular Biology Graduate Program, University of Massachusetts Amherst, Amherst, MA, United States

**Keywords:** bacterial gene regulation, CRISPR interference (CRISPRi), CRISPR activation (CRISPRa), transcriptional interference, transcriptional activation, non-model bacteria, genome-wide library

## Abstract

CRISPR interference (CRISPRi) and CRISPR activation (CRISPRa) have become ubiquitous approaches to control gene expression in bacteria due to their simple design and effectiveness. By regulating transcription of a target gene(s), CRISPRi/a can dynamically engineer cellular metabolism, implement transcriptional regulation circuitry, or elucidate genotype-phenotype relationships from smaller targeted libraries up to whole genome-wide libraries. While CRISPRi/a has been primarily established in the model bacteria *Escherichia coli* and *Bacillus subtilis*, a growing numbering of studies have demonstrated the extension of these tools to other species of bacteria (here broadly referred to as non-model bacteria). In this mini-review, we discuss the challenges that contribute to the slower creation of CRISPRi/a tools in diverse, non-model bacteria and summarize the current state of these approaches across bacterial phyla. We find that despite the potential difficulties in establishing novel CRISPRi/a in non-model microbes, over 190 recent examples across eight bacterial phyla have been reported in the literature. Most studies have focused on tool development or used these CRISPRi/a approaches to interrogate gene function, with fewer examples applying CRISPRi/a gene regulation for metabolic engineering or high-throughput screens and selections. To date, most CRISPRi/a reports have been developed for common strains of non-model bacterial species, suggesting barriers remain to establish these genetic tools in undomesticated bacteria. More efficient and generalizable methods will help realize the immense potential of programmable CRISPR-based transcriptional control in diverse bacteria.

## Introduction

Since the development of CRISPR interference (CRISPRi) (Qi et al., 2013) and CRISPR activation (CRISPRa) (Bikard et al., 2013) in 2013, they have become efficient and prevalent tools for transcriptional regulation in bacteria. CRISPR-Cas originates as a form of prokaryotic immunity, with systems comprising one or more CRISPR-associated (Cas) proteins and a short guide RNA (gRNA) that complex together to target and cleave foreign DNA or RNA molecules, such as viruses (Nussenzweig and Marraffini, 2020). The gRNA leads the complex to target sequence *via* complementarity between the protospacer sequence of the gRNA and the target site on the DNA/RNA molecule. Various mechanisms exist to prevent cleavage of chromosomal DNA, which most often involves a protospacer adjacent motif (PAM) or equivalent next to the target site that is not present in the CRISPR arrays on the chromosome (Jackson et al., 2017).

Researchers developed CRISPRi technology by deactivating the nuclease activity of select Cas enzymes to create mutant dCas proteins that bind, but do not cleave, the DNA target (Qi et al., 2013). Most CRISPRi systems repress a gene’s expression through steric inhibition of RNA polymerase binding or extension (Qi et al., 2013), although some repress gene expression through RNA cleavage (Zhang K. et al., 2020; Rahman et al., 2021). Gene repression over 100-fold has been reported for several diverse CRISPRi tools and can approach near knockout levels of gene expression (Qi et al., 2013; Miao et al., 2019). Targeting a different sequence is easily achieved by changing the short protospacer sequence on the gRNA to bind a location within the promoter, untranslated region, or coding sequence of the target gene based on simple design rules (Qi et al., 2013; Zetsche et al., 2015; Zhang et al., 2017). Additionally, multiplexed gene repression can be achieved by simply expressing multiple gRNA within a cell (Qi et al., 2013; Zhang et al., 2017).

Shortly after the development of CRISPRi, researchers developed CRISPRa for bacterial transcriptional activation by combining the dCas protein with a transcriptional activator that recruits transcription machinery to the target gene’s promoter to increase gene expression (Bikard et al., 2013). The specific mechanism for transcriptional activation depends on the activator, which can be incorporated by directly fusing a transcriptional activator domain to the dCas protein (Bikard et al., 2013; Ho et al., 2020; Schilling et al., 2020), incorporating RNA scaffolds into the gRNA sequence to recruit activator domains to the dCas complex (Dong et al., 2018; Liu Y. et al., 2019; Fontana et al., 2020a), or using non-covalent protein-protein interaction domains to complex the transcriptional activator and dCas protein (Villegas Kcam et al., 2021; Villegas Kcam et al., 2022). Unlike CRISPRi, however, CRISPRa has complex design rules that often strongly depend on the CRISPRa technology (i.e., type of activation domain and approach to couple the activation domain and dCas complex) as wells as several other factors (Liu Y. et al., 2019; Fontana et al., 2020a; Ho et al., 2020; Villegas Kcam et al., 2021). These other considerations include the basal expression of the target gene and the location of the binding site for the CRISPRa complex, where activation is typically achieved in a narrow range upstream of the target gene’s promoter and the activation strength fluctuates sharply as the nucleotide position shifts. Combined with the PAM requirement for DNA binding, these requirements greatly restrict the available DNA target sites for effective gene activation, especially for endogenous genes. Due to these relatively stringent design rules for gene activation and often low (<10-fold) activation levels compared to CRISPRi repression (Bikard et al., 2013; Liu W. et al., 2019; Fontana et al., 2020a; Villegas Kcam et al., 2021), CRISPRa development has been slower in bacteria than in eukaryotes (Kampmann, 2018; Fontana et al., 2020b). Despite the current limitations of CRISPRa, however, the simplicity and inherent properties of CRISPRi/a gene regulation can provide strong transcriptional control of multiple genes simultaneously, making these approaches often easier and faster than traditional methods and allowing for dynamic transcriptional control.

CRISPR systems are classified into a variety of classes, types, subtypes, and variants, each with unique genes and properties (Koonin et al., 2017; Makarova et al., 2020). Many systems have been engineered to create effective CRISPRi/a tools. The first and most common tool is derived from the Type II Cas9 system, which comprises a single deactivated Cas9 (dCas9) protein and two small RNAs that create the gRNA (Qi et al., 2013). These two RNAs can be combined into a synthetic single guide RNA (sgRNA) for easier synthesis, but each sgRNA requires an independent promoter for expression (Jiang et al., 2015). Although many different dCas9 variants exist, the *Streptococcus pyogenes* dCas9 (Sp dCas9) system is the most common due to its short PAM sequence and strong transcription regulation abilities. Recently, tools derived from the Type V Cas12a (formerly Cpf1) system have been developed, which uses a single deactivated Cas12a (dCas12a) protein and one gRNA (Zetsche et al., 2015; Kim et al., 2017; Zhang et al., 2017; Miao et al., 2019). Unlike dCas9, dCas12a can process its gRNA from CRISPR arrays, providing easier multiplexed regulation (Fonfara et al., 2016; Zhang et al., 2017). Additionally, several studies have suggested that dCas12a variants are less toxic than dCas9 variants across different bacterial phyla (Liu W. et al., 2019; Knoot et al., 2020; Kuo et al., 2020), making them an attractive alternative to dCas9. The most common dCas12a variants used in bacteria are derived from *Francisella tularensis* subsp. *novicida* (Fn dCas12a) and *Acidaminococcus* sp. BV3L6 (As dCas12a). Several Type I CRISPRi/a tools have been designed, but due to the large number of genes in these systems, most tools are implemented by reprogramming the host species’ endogenous CRISPR system for gene repression (Luo et al., 2015; Xu et al., 2021; Villegas Kcam et al., 2022). Only a handful of CRISPRi tools from other systems have been reported for transcriptional regulation in bacteria, likely due to the novelty of the system (Rahman et al., 2021) or high cellular toxicity observed upon expression (Zhang K. et al., 2020).

Despite the unique traits and relevance of a vast diversity of bacteria, CRISPRi/a tools have been primarily developed in the model bacteria *Escherichia coli* and *Bacillus subtilis*. Yet, non-model bacteria (a broad definition of non-model, excluding *E. coli* and *B. subtilis*, is used here) offer great promise in research and industry spanning a wide range of medical, environmental, and biomanufacturing applications. For example, *Streptomyces*, *Sorangium*, and *Photorhabdus* spp. naturally produce bioactive secondary metabolites, such as antibiotics, and contain silent biosynthetic gene clusters with unknown and potentially useful products (Ye et al., 2019; Tian et al., 2020; Ke et al., 2021). Additionally, *Rhodococcus* and *Corynebacterium* spp. can produce valuable chemicals from cheap and simple feedstock and are tolerant to harsh conditions, making them ideal cell factories (Cleto et al., 2016; DeLorenzo et al., 2018). However, several conditions must be reached to successfully establish efficient CRISPRi/a tools in a non-model bacterium. In this mini-review, we detail these criteria, emphasizing the importance of characterized genetic parts to tightly control the expression of CRISPRi/a systems to limit potential toxicity while providing sufficient expression for effective transcriptional control. We demonstrate that despite the potential difficulties in creating these tools in non-model bacteria, they have been established across eight different bacterial phyla and have been used for a variety of applications, including high-throughput genome-wide selections. Finally, we highlight the current challenges to developing CRISPRi/a tools in non-model bacteria and novel species, which suggest directions for future progress.

## Requirements and Challenges to Establish CRISPRi/a in Non-Model Bacteria

Several criteria must be met to successfully establish an effective CRISPRi/a tool in a non-model species or strain. First, the conditions for culturing, maintaining, and genetically manipulating the strain (often referred to as strain “domestication”) must be determined. For a phylogenetically similar strain to a previously established model bacteria, such as many *Bacillus* species (Zhan et al., 2020) and Enterobacteriaceae (Ho et al., 2020), suitable culture conditions may be similar to those previously determined. For novel or fastidious species, however, trial and error and patience may be required to determine appropriate culture conditions for growth and genetic manipulation, such as the obligate intracellular pathogen *Chlamydia trachomatis* (Ouellette, 2018). Additionally, introducing foreign DNA is often challenging for a non-model bacterium, as many are genetically recalcitrant, especially pathogens (Fernandes et al., 2021b) and novel strains (Zhao et al., 2020; Jin et al., 2022), and establishing a sufficient genetic transformation method can require significant effort. Additionally, care must be taken when introducing synthetic DNA to circumvent the bacterial host’s native immunity that may degrade foreign DNA, including restriction-modification and CRISPR systems (Marraffini and Sontheimer, 2008; Jin et al., 2022), such as by mimicking the recipient strain’s methylation patterns (Monk et al., 2015; Zhao et al., 2020). More discussion on the isolation and domestication of non-model bacteria can be found in other reviews (Vartoukian et al., 2010; Lewis et al., 2021; Riley and Guss, 2021).

Next, reliable genetic parts for the non-model bacterium are required to be able to express and tightly control the CRISPRi/a tool, including promoters, ribosome binding sites, terminators, and expression or integration vectors. For many non-model bacteria, especially novel species, these genetic part libraries are unavailable, and so, the necessary genetic parts must be created and characterized. In some cases, established genetic parts may be transferable from a model bacterium to a related species, such as promoters between Gram-positive bacteria (Liew et al., 2010). However, genetic parts often do not function equivalently between bacterial species or even strains (Tong et al., 2015; Leonard et al., 2018). Each CRISPRi/a component should be expressed using unique genetic parts to prevent repeated DNA sequences. Since dCas protein expression can elicit cytotoxicity, high strength promoters used for overexpression may not be optimal. If existing genetic parts are insufficient for a new bacterial species, identifying genetic regulatory elements from the endogenous genome provides an alternative to synthetic DNA design strategies (Fernandes et al., 2019). Libraries of genetic parts and inducible promoters are excellent tools to tune the expression of CRISPRi/a systems, and several studies have established such toolboxes in non-model bacteria to facilitate the development of genetic tools such as CRISPRi/a (Mimee et al., 2015; Leonard et al., 2018; Shin et al., 2019; Teh et al., 2019; Liow et al., 2020). These libraries and tunable parts are especially important to control the expression of the CRISPRi/a tool to minimize potential cellular toxicity and to precisely control transcriptional regulation (Qu et al., 2019; Bosch et al., 2021; Shabestary et al., 2021).

In the design of a synthetic CRISPRi/a system for a bacterium, consideration should be given to prevent interference with endogenous CRISPR systems and/or anti-CRISPR genes harbored on the strain’s genome. If the foreign and native CRISPR-Cas types are too similar, the introduction of the synthetic gRNA may induce cleavage of the host bacterium’s genome (*via* the catalytically active endogenous Cas enzyme) and can cause cell death in a DNA repair-deficient strain or undesired mutations if the strain has appropriate DNA repair pathways. This can be avoided by choosing a CRISPRi/a tool that does not share significant homology to any endogenous CRISPR-Cas. Native CRISPR-Cas systems can be predicted from the sequenced genome or proteome using computer software (Couvin et al., 2018; Chai et al., 2019), aiding in CRISPRi/a tool selection for novel strains. Alternatively, the native system can be engineered to create a CRISPRi/a tool *via* genetic manipulation, such as the deletion of the native *cas2/3* or *cas3* gene responsible for cleavage in Type I-F systems (Zheng et al., 2019; Qin et al., 2021; Xu et al., 2021) or mutating the native *cas9* sequence for Type II systems (Shields et al., 2020; Dammann et al., 2021). Anti-CRISPR proteins, which inhibit CRISPR systems through a variety of mechanisms (Pawluk et al., 2018), may require deletion or disruption before a heterologous CRISPRi/a tool can be expressed (Xu et al., 2021). Online tools and databases are available to predict and describe anti-CRISPR proteins from protein sequences to help select an appropriate CRISPRi/a system (Wang et al., 2020; Wang et al., 2021a).

Finally, the CRISPRi/a components should be expressed at a level that provides adequate transcriptional repression or activation for the given application without significant cellular toxicity. Many studies have reported CRISPRi toxicity for diverse bacteria, while little is known about CRISPRa toxicity due to limited reports in the literature. These observed forms of toxicity include changes in cell morphology (Cho et al., 2018; Ouellette et al., 2021) and slower growth or complete growth inhibition (Rock et al., 2017; Yu et al., 2018; Wurihan et al., 2019; Zhang K. et al., 2020; Brito et al., 2020). To prevent toxicity, one can use a less toxic CRISPRi/a system for the host species (Rock et al., 2017; Zhao et al., 2019), or reduce the expression of the components by substituting genetic parts (Qu et al., 2019). Expansive libraries of genetic parts, including inducible and constitutive promoters, ribosome binding sites, and protein degradation tags, can be used to tune gene expression and characteristics of the CRISPRi/a tool (Depardieu and Bikard, 2020; Fleck and Grundner, 2021; Ouellette et al., 2021). However, the components cannot be expressed so low that it cannot effectively repress or activate the target gene(s), especially during multiplexed gene regulation that relies on a shared dCas protein pool for multiple gRNAs (Zhang and Voigt, 2018; Zhao et al., 2018). A careful balance is required to express the CRISPRi/a components.

## Current CRISPRi/a Tools for Non-Model Bacteria

Different CRISPRi/a tools have been established in a range of bacteria that span many phyla and have been used for a variety of applications, as summarized here (Table 1). Overwhelmingly, these studies have utilized the Sp dCas9 CRISPRi system. More detailed information for each study can be found in Supplementary Table S1 (Supplementary Data Sheet 1).

**TABLE 1:** CRISPRi/a studies in non-model bacteria and their key characteristics.

Bacterium	Application	CRISPRi/a	CRISPR System(s)^a^	GW^b^	References^c^ and type of study
**Actinomycetota**					
*Bifidobacterium* spp.	Probiotic	CRISPRi	As dCas12a	N	TD: Jin et al. (2022)
*Corynebacterium glutamicum*	Bioproduction	CRISPRa	Fn dCas12a- ω	N	TD: Liu et al. (2019a)
		CRISPRi	Fn dCas12a	N	TD: Liu et al. (2019a); Li et al. (2020b); ME: Liu et al. (2019a); Li et al. (2020b); Huang et al. (2021)
			Sp dCas9	N	TD: Cleto et al. (2016); Zhang et al. (2016); Park et al. (2018); Gauttam et al. (2019); MGF: Li and Liu, (2017); Lee et al. (2018); ME: Cleto et al. (2016); Zhang et al. (2016); Park et al., 2018, 2019; Yoon and Woo, (2018); Gauttam et al. (2019)
				N*	SS: Göttl et al. (2021)
			Rf Cas13d	N	TD: Zhang et al. (2020a)
***Mycobacterium***					
*M. smegmatis, M. tuberculosis*	Pathogen	CRISPRi	Fn dCas12a	N	TD: Fleck and Grundner, (2021)
*M. smegmatis, M. tuberculosis, M. bovis*	Pathogen	CRISPRi	Sp dCas9	N	TD: Choudhary et al. (2015); Singh et al. (2016); Xiao et al. (2019); Agarwal, (2020); Nadolinskaia et al. (2021); MGF: Thakur et al. (2016); Singh et al. (2017); Choudhary et al. (2019); Dutta et al. (2019); Agarwal, (2020); Lunge et al. (2020); Faulkner et al. (2021); Gani et al. (2021); Gibson et al. (2021)
*M. smegmatis, M. tuberculosis*	Pathogen	CRISPRi	Sth1 dCas9	N	TD: Rock et al. (2017); Cheung et al. (2021); Judd et al. (2021); MGF: Baranowski et al. (2018); Landeta et al. (2019); Mai et al. (2019); McNeil and Cook, (2019); McNeil et al. (2020); McNeil et al. (2022); Randall et al. (2020); Brzostek et al. (2021); Quiñones-Garcia et al. (2021); Savková et al. (2021)
				N*	SS: de Wet et al. (2020); McNeil et al. (2021)
				Y	SS: Bosch et al. (2021)
*M. tuberculosis*	Pathogen	CRISPRi	Native Type III-A	Y	TD: Rahman et al. (2021); SS: Rahman et al. (2021)
*Rhodococcus opacus*	Bioproduction	CRISPRi	Sth1 dCas9	N	TD: DeLorenzo et al., 2018, 2021; ME: DeLorenzo et al. (2018)
*Saccharopolyspora erythraea*	Bioproduction, bioresearch	CRISPRi	Sp dCas9	N	ME: Liu et al. (2021b)
***Streptomyces***					
*S. venezuelae*	Bioproduction, bioresearch	CRISPRa	Sp dCas9-αNTD	N	TD: Ameruoso et al. (2021)
*S. coelicolor*	Bioproduction, bioresearch	CRISPRi	Fn dCas12a	N	TD: Li et al. (2018); MGF: Yan et al. (2022); ME: Liu et al. (2021c)
*S. coelicolor, S. venezuelae, S. rapamycinicus, S.* spp.	Bioproduction, bioresearch	CRISPRi	Sp dCas9	N	TD: Tong et al. (2015); Tong et al. (2020); Zhao et al. (2018); Tian et al. (2020); Ameruoso et al. (2021); Wang et al. (2021b); ME: Tian et al. (2020); MGF: Ultee et al. (2020); Zhang et al. (2020b); Zhang et al. (2021); TRN: Tian et al. (2020)
**Bacteroidetes**					
*Bacteroides thetaiotaomicron*	Probiotic	CRISPRi	Sp dCas9	N	TD: Mimee et al. (2015); TRN: Mimee et al. (2015); Taketani et al. (2020)
*Bacteroides, Parabacteroides, Prevotella* spp.	Probiotic	CRISPRi	As dCas12a	N	TD: Jin et al. (2022)
**Chlamydiae**					
*Chlamydia trachomatis*	Pathogen	CRISPRi	As dCas12a	N	TD: Ouellette et al. (2021)
			Sa dCas9	N	TD: Ouellette, (2018); Wurihan et al. (2019); Ouellette et al. (2021); MGF: Brockett et al. (2021)
**Cyanobacteria**					
*Anabaena* sp. PCC 7120	Bioproduction, bioresearch	CRISPRi	Sp dCas9	N	TD: Higo et al. (2018); Higo and Ehira, (2019); ME: Higo et al. (2018); Higo and Ehira, (2019); MGF: Higo et al. (2019)
*Synechococcus* sp. UTEX 2973	Bioproduction	CRISPRi	Fn dCas12a	N	TD: Knoot et al. (2020); MGF: Knoot et al. (2020)
*Synechococcus elongatus*	Bioproduction	CRISPRi	Fn dCas12a	N	TD: Choi and Woo, (2020); ME: Choi and Woo, (2020)
			Sp dCas9	N	TD: Huang et al. (2016); ME: Huang et al. (2016); TRN: Lee and Woo, (2020)
*Synechococcus* sp. PCC 7002	Bioproduction	CRISPRi	Sp dCas9	N	TD: Gordon et al. (2016); ME: Gordon et al. (2016)
*Synechocystis* sp. PCC 6803	Bioproduction, bioresearch	CRISPRi	Fn dCas12a	N	TD: Liu et al. (2020a); MGF: Liu et al. (2020a)
			Sp dCas9	N	TD: Yao et al. (2016); Kirtania et al. (2019); MGF: Behler et al. (2018); Kaczmarzyk et al. (2018); Behle et al. (2021); 3; Santos et al. (2021); Shabestary et al. (2021); ME: Kaczmarzyk et al. (2018); Shabestary et al. (2018); Shabestary et al. (2021); Dietsch et al. (2021); Yunus et al. (2022)
				Y	SS: Yao et al. (2020)
**Firmicutes**					
***Bacillus***					
*B. amyloliquefaciens*	Bioproduction	CRISPRa	Sp dCas9-ω	N	TD: Zhao et al. (2020); ME: Zhao et al. (2020)
*B. amyloliquefaciens, B. methanolicus, B. licheniformis*	Bioproduction	CRISPRi	Sp dCas9	N	TD: Schultenkämper et al. (2019); Sha et al. (2020); Zhan et al. (2020); Zhao et al. (2020); MGF: Schultenkämper et al. (2019); Schultenkämper et al. (2021); ME: Sha et al. (2020); Zhan et al. (2020)
*B. smithii*	Bioproduction	CRISPRi	ThermodCas9	N	TD: Mougiakos et al. (2017)
*Clostridioides difficile*	Pathogen	CRISPRi	Sp dCas9	N	TD: Marreddy et al. (2019); Müh et al. (2019); MGF: Marreddy et al. (2019); Müh et al. (2019)
***Clostridium***					
*C. sporogenes, C.* spp.	Probiotic	CRISPRi	As dCas12a	N	TD: Jin et al. (2022)
*C. ljungdahlii*	Bioproduction	CRISPRi	Fn dCas12a	N	TD: Zhao et al. (2019); ME: Zhao et al. (2019)
*C. autoethanogenum, C. acetobutylicum, C. beijerinckii, C. pasteurianum, C. cellulovorans, C. ljungdahlii*	Bioproduction	CRISPRi	Sp dCas9	N	TD: Bruder et al. (2016); Li et al. (2016); Wang et al. (2016); Wen et al. (2017); Woolston et al. (2018); Fackler et al. (2021); ME: Wen et al. (2017); Woolston et al. (2018)
*Enterococcus faecalis*	Pathogen	CRISPRi	Sp dCas9	N	TD: Peters et al. (2019); Afonina et al. (2020); MGF: Afonina et al. (2020)
*Eubacterium limosum*	Bioproduction, probiotic	CRISPRi	Sp dCas9	N	TD: Shin et al. (2019)
*Hungateiclostridium thermocellum*	Bioproduction	CRISPRi	ThermodCas9	N	TD: Ganguly et al. (2020)
*Lactiplantibacillus plantarum*	Probiotic, bioproduction	CRISPRi	Sp dCas9	N	TD: Myrbråten et al. (2019)
					MGF: Myrbråten et al. (2019)
*Lactococcus lactis*	Probiotic, bioproduction	CRISPRi	Sp dCas9	N	TD: Berlec et al. (2018); Xiong et al. (2020)
*Leuconostoc citreum*	Probiotic	CRISPRi	Sp dCas9	N	TD: Son et al. (2020)
					ME: Son et al. (2020)
*Listeria monocytogenes*	Pathogen	CRISPRi	Sp dCas9	N	TD: Peters et al. (2019)
*Paenibacillus polymyxa*	Bioproduction	CRISPRa	As dCas12a-SoxS	N	TD: Schilling et al. (2020); ME: Schilling et al. (2020)
*Paenibacillus sonchi*	Plant symbiote	CRISPRi	Sp dCas9	N	TD: Brito et al. (2020)
*Staphylococcus aureus, S. epidermidis*	Pathogen	CRISPRi	Sp dCas9	N	TD: Chen et al. (2017); Dong et al. (2017); Zhao et al. (2017); Sato’o et al. (2018); Stamsås et al. (2018); Peters et al. (2019); Depardieu and Bikard, (2020); Jiang et al. (2020); Spoto et al. (2020); MGF: Wang and Nicholaou, (2017); Stamsås et al. (2018); Wu et al. (2019); Gelin et al. (2020); Mårli, (2020); Gallay et al. (2021); Myrbråten et al. (2021); Wang and Sun, (2021a)
				Y	SS: Jiang et al. (2020); Mårli, (2020); Spoto et al. (2021)
***Streptococcus***					
*S. pneumoniae, S. salivarius*	Pathogen	CRISPRi	Sp dCas9	N	TD: Bikard et al. (2013); Liu et al. (2017); MGF: Domenech et al. (2018); Gallay et al. (2021); Knoops et al. (2022)
*S. pneumoniae*	Pathogen	CRISPRi	Sp dCas9	N*	SS: Liu et al. (2017)
				Y	SS: Dewachter et al. (2021); Gallay et al. (2021); Liu et al. (2021a); de Bakker et al. (2022)
*S. agalactiae*	Pathogen	CRISPRi	Native dCas9	N	TD: Dammann et al. (2021); MGF: Dammann et al. (2021)
*S. mutans*	Pathogen	CRISPRi	Native dCas9	N*	TD: Shields et al. (2020); SS: Shields et al. (2020)
**Proteobacteria**					
*Acidithiobacillus ferrooxidans*	Bioresearch, bioremediation	CRISPRi	Sp dCas9	N	TD: Yamada et al. (2022)
*Acinetobacter baumannii, A. baylyi*	Pathogen	CRISPRi	Sp dCas9	N	TD: Geng et al. (2019); Peters et al. (2019); Bai et al. (2021); MGF: Bai et al. (2021); Colquhoun et al. (2021); Dai et al. (2021)
*Aeromonas hydrophila*	Bioproduction, bioresearch, bioremediation	CRISPRi	Sp dCas9	N	TD: Wu et al. (2020); MGF: Wu et al. (2020)
*Bartonella apis*	Bee probiotic	CRISPRi	Sp dCas9	N	TD: Leonard et al. (2018)
*Burkholderia cenocepacia, B. multivorans, B.thailandensis*	Pathogen	CRISPRi	Sp dCas9	N	TD: Hogan et al. (2019)
*Caulobacter crescentus*	Bioresearch	CRISPRi	Spa dCas9, Sth1 dCas9	N	TD: Guzzo et al. (2020)
			Sp dCas9	N	TD: Irnov et al. (2017); MGF: Irnov et al. (2017); Werner et al. (2020)
*Chromobacterium violaceum*	Biorecovery	CRISPRi	Sp dCas9	N	TD: Liow et al. (2020)
*Enterobacter cloacae*	Pathogen	CRISPRi	Sp dCas9	N	TD: Peters et al. (2019)
*Gluconobacter oxydans*	Bioproduction	CRISPRi	Native Type I-E	N	TD: Qin et al. (2021)
*Halomonas* sp. TD01	Bioproduction	CRISPRi	Sp dCas9	N	TD: Tao et al. (2017); ME: Tao et al. (2017)
***Klebsiella***					
*K. oxytoca*	Pathogen	CRISPRa	Sp dxCas9	N	TD: Liu et al. (2019b)
			Sp dCas9-AsiA v2.1	N	TD: Ho et al. (2020)
*K. pneumoniae, K.oxytoca, K. aerogenes*	Pathogen, bioproduction	CRISPRi	Sp dCas9	N	TD: Wang et al. (2018a); Peters et al. (2019); Ho et al. (2020); ME: Wang et al. (2017); Wang et al. (2018a)
*Komagataeibacter hansenii, K. xylinus*	Bioproduction	CRISPRi	Sp dCas9	N	TD: Teh et al. (2019); Huang et al. (2020); MGF: Huang et al. (2020); ME: Huang et al. (2020)
*Legionella pneumophila*	Pathogen	CRISPRi	Sp dCas9	N	TD: Ellis et al. (2021); MGF: Ellis et al. (2021)
*Lysobacter enzymogenes*	Bioproduction, bioresearch	CRISPRa	Sp dCas9-ω	N	TD: Yu et al. (2018); 11; ME: Yu et al. (2018); 11
*Methylorubrum extorquens*	Bioproduction	CRISPRi	Sp dCas9	N	TD: Mo et al. (2020); MGF: Mo et al. (2020); ME: Mo et al. (2020)
*Myxococcus xanthus*	Bioproduction	CRISPRa	Sp dCas9-ω	N	TD: Peng et al. (2018); Wang et al. (2021c); MGF: Peng et al. (2018); Wang et al. (2021c); ME: Peng et al. (2018); Wang et al. (2021c)
*Photorhabdus luminescens*	Bioresearch	CRISPRa	Sp dCas9-ω	N	TD: Ke et al. (2021); MGF: Ke et al. (2021)
*Proteus mirabilis*	Pathogen	CRISPRi	Sp dCas9	N	TD: Peters et al. (2019)
***Pseudomonas***					
*P. putida*	Bioproduction, bioremediation	CRISPRa/i	Sp dCas9+MCP	N	TD: Kiattisewee et al. (2021); ME: Kiattisewee et al. (2021)
		CRISPRi	Fn dCas12a	N	ME: Banerjee et al. (2020); Czajka et al. (2021)
*P. putida, P. fluorescens*	Bioproduction, plant symbiote, bioremediation	CRISPRi	Spa dCas9	N	TD: Tan et al. (2018); MGF: Gauttam et al. (2021)
			Sp dCas9	N	TD: Sun et al. (2018); Noirot-Gros et al. (2019); Batianis et al. (2020); Kim et al. (2020); MGF: Noirot-Gros et al. (2019); ME: Kim et al. (2020); Kozaeva et al. (2021); Li and Ye, (2021); TRN: Liu et al. (2020b)
*P. aeruginosa*	Pathogen	CRISPRi	Spa dCas9	N	TD: McMackin et al. (2019); Gauttam et al. (2021); MGF: McMackin et al. (2019); Gauttam et al. (2021)
			Sp dCas9	N	TD: Peters et al. (2019); Xiang et al. (2020); Stolle et al. (2021)
			Sp dCas9, Sth1 dCas9	N	TD: Qu et al. (2019); MGF: Qu et al. (2019)
			Native Type I-F	N	TD: Xu et al. (2021)
*Rhodobacter capsulatus*	Bioproduction	CRISPRi	Fn dCas12a	N	TD: Zhang and Yuan, (2021)
*Salmonella enterica*	Pathogen	CRISPRa/i	Sp dCas9-ω	N	TD: Bhokisham et al. (2020); TRN: Bhokisham et al. (2020)
		CRISPRa	Sp dCas9-AsiA v2.1	N	TD: Ho et al. (2020)
		CRISPRi	Sp dCas9	N	TD: Peters et al. (2019); Ho et al. (2020)
			Ec Type I-E	N	TD: Rath et al. (2015)
*Shewanella oneidensis*	Bioproduction, bioresearch	CRISPRi	As dCas12a	N	TD: Li et al. (2020a); MGF: Li et al. (2020a)
		CRISPRi	Sp dCas9	N	TD: Cao et al. (2017); ME: Yi and Ng, (2021)
*Sorangium cellulosum*	Bioresearch	CRISPRa	Sp dCas9-VP64	N	TD: Ye et al. (2019); MGF: Ye et al. (2019)
*Vibrio casei*	Bioproduction	CRISPRi	Sp dCas9	N	TD: Peters et al. (2019)
*Vibrio natriegens*	Bioproduction	CRISPRi	Sp dCas9	Y	TD: Lee et al. (2019); SS: Lee et al. (2019)
*Vibrio cholerae*	Pathogen	CRISPRi	Sp dCas9	N	TD: Caro et al. (2019); Wiles et al. (2020); MGF: Caro et al. (2019); Wiles et al. (2020)
*Yersinia pestis*	Pathogen	CRISPRi	Sp dCas9	N	TD: Wang et al. (2019)
*Zymomonas mobilis*	Bioproduction	CRISPRi	Sp dCas9	N	TD: Banta et al. (2020); MGF: Banta et al. (2020)
			Native Type I-F	N	TD: Zheng et al. (2019)
**Spirochaetes**					
*Borreliella burgdorferi*	Pathogen	CRISPRi	Sp dCas9	N	TD: Takacs et al. (2020)
*Leptospira interrogans, L. biflexa, L.* strain LGVF02	Pathogen	CRISPRi	Sp dCas9	N	TD: Fernandes et al. (2019); Fernandes et al. (2021a); Fernandes et al. (2021b); MGF: Fernandes et al. (2021b)
**Tenericutes**					
*Mycoplasma pneumoniae*, *M.* JCVI-syn1.0*, M.* JCVI-syn3.0	Synthetic cells	CRISPRi	Sp dCas9	N	TD: Mariscal et al. (2018)

a Acronyms for each CRISPR system can be found in Supplementary Table S2 (Supplementary Data Sheet 1).

b Genome-wide (GW) classification for the relative size of the gRNA library: yes (Y) indicates a genome-wide library targeting >90% of coding genes on the genome; no (N) indicates a library of <50 target genes; and a smaller library targeting >50 genes but <90% of genome is indicated (N*).

c Classifications for types of studies: tool development (TD), mapping gene function (MGF), metabolic engineering (ME), screens and/or selections (SS), transcriptional regulatory network (TRN).

### Actinomycetota

CRISPRi has been well established in a wide range of Actinobacteria, including *Mycobacteria*, *Streptomyces*, and *Corynebacterium*, and has been used for metabolic engineering and the elucidation of gene functions in both small studies and genome-wide screens (Table 1). Additionally, several CRISPRi tools are commonly used in *Mycobacteria* (Choudhary et al., 2015; Rock et al., 2017; Agarwal, 2020; Fleck and Grundner, 2021) and *Streptomycetes* (Tong et al., 2015; Li et al., 2018; Zhao et al., 2018). CRISPRa has also been recently establish in *Corynebacterium* (Liu W. et al., 2019) and *Streptomycetes* (Ameruoso et al., 2021).

### Cyanobacteria

CRISPRi/a is especially useful in cyanobacteria due to their polyploidal genomes (Kirtania et al., 2019). CRISPRi is relatively well-established in a wide range of cyanobacterial species, including those of research and industrial significance, and has been used for metabolic engineering, transcriptional regulatory networks, and the study of gene functions in small studies and a genome-wide screen/selection (Table 1). Many CRISPRi tools are available in cyanobacteria, each with their own characteristics (Gordon et al., 2016; Yao et al., 2016; Liu D. et al., 2020; Choi and Woo, 2020). CRISPRa has not yet been reported in cyanobacteria.

### Firmicutes

CRISPRi is well-established in a wide range of Firmicutes, including *Bacilli*, *Clostridia*, *Staphylococci*, and *Streptococci* (Table 1). CRISPRi tools have been developed and used for metabolic engineering, elucidation of gene functions, and genome-wide screens and selections. CRISPRa has been reported in *Bacillus amyloliquefaciens* and *Paenibacillus polymyxa* in tool development work and some metabolic engineering applications (Schilling et al., 2020; Zhao et al., 2020).

### Proteobacteria

CRISPRi and CRISPRa are well established in a wide variety of Proteobacteria, including *Klebsiella, Salmonella*, *Pseudomonas*, and *Vibrio* (Table 1). These tools have been developed and used for metabolic engineering, synthetic transcriptional regulatory networks, and mapping gene function using small gRNA sets and genome-wide screens and selections. Reports of CRISPRi are far more common than CRISPRa.

### Other Bacterial Phyla

CRISPRi has also been reported in the phyla Chlamydiae, Tenericutes, Spirochaetes, and Bacteroidetes (Table 1). Although these reports have primarily been for tool development, some have used CRISPRi to investigate gene function (Fernandes et al., 2021b; Brockett et al., 2021) or create synthetic genetic circuits (Mimee et al., 2015; Taketani et al., 2020).

## Applications of CRISPRi/a in Non-Model Bacteria

**FIGURE 1:**
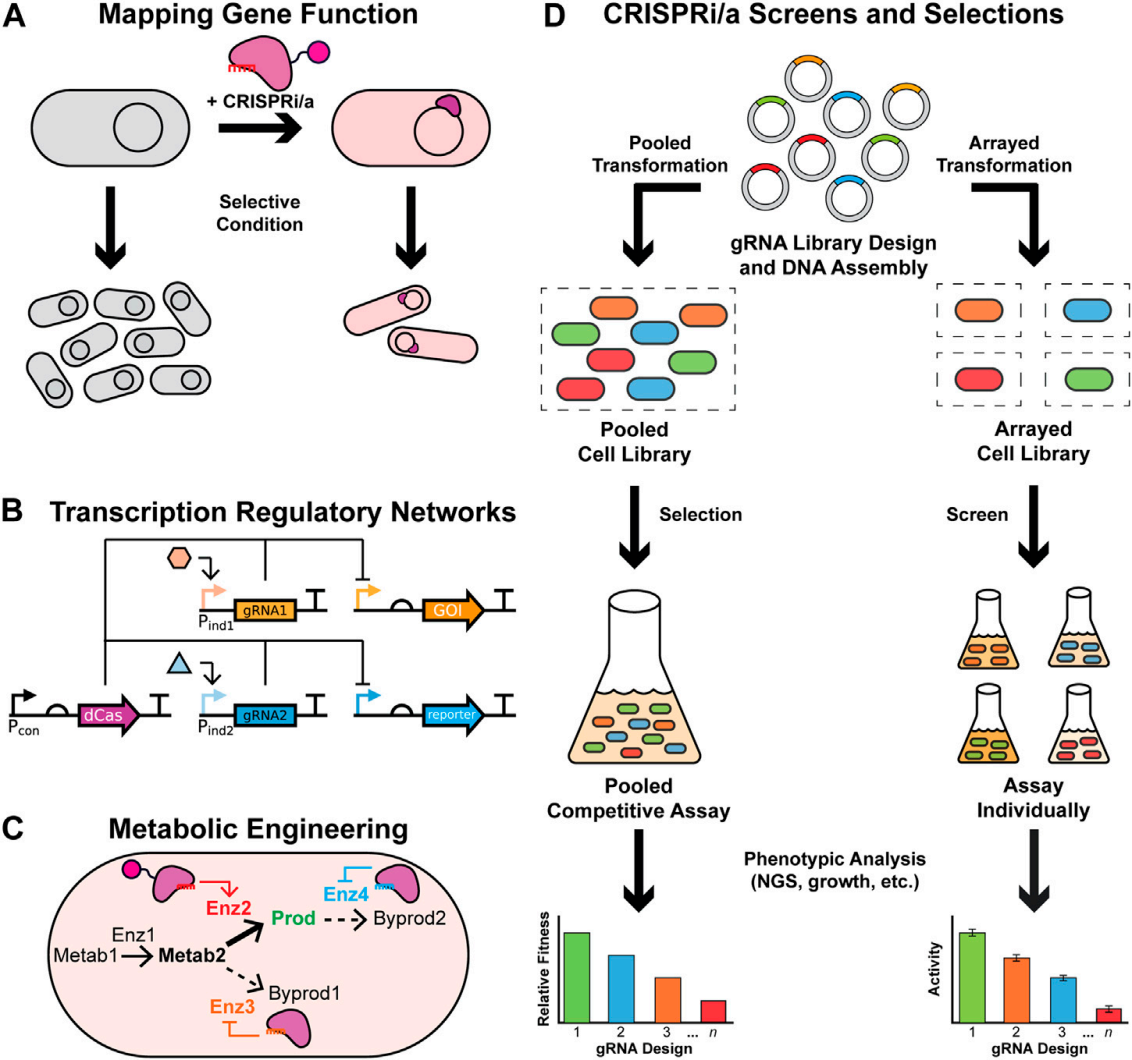
Applications of CRISPRi/a tools in bacteria. **(A)** CRISPRi/a tools can be used to study gene function by targeting a gene for repression or activation and assaying phenotypic differences under a given condition. **(B)** CRISPRi/a can control transcription regulatory networks through the expression of multiple guide RNAs and a single deactivated Cas (dCas) protein. By incorporating inducible promoters for the guide RNAs (gRNAs) and/or dCas protein, dynamic genetic circuits can be created that respond to multiple input stimuli to alter the expression of output genes, such as a gene of interest (GOI) and a reporter gene. **(C)** CRISPRi/a can be used for dynamic metabolic engineering by regulating gene expression to redirect carbon flux towards production of a desired biochemical, such as using CRISPRi to repress genes encoding competing enzymes (dashed arrows) while using CRISPRa to activate genes for the desired biochemical production (bold arrow). **(D)** CRISPRi/a has been used for high-throughput, large-scale screens and selections ranging from targeted sets of genes to genome-wide libraries. After designing and assembling the gRNA library for the given application, the library is transformed into bacteria that express the cognate dCas protein in either a mixed pooled (i.e., cells containing different gRNA(s) are mixed together) or an individual array format (i.e., cells containing different gRNA(s) are cultured separately). The resulting pooled cell library can be used in competitive selections for functional genomics analyses by applying selective conditions and measuring the relative gRNA fitness. An arrayed cell library can be assayed one-by-one to determine the relationship between strain activity (e.g. production or phenotype) and gene repression/activation.

CRISPRi/a tools can be used for a variety of applications in non-model bacteria (Figure 1). The most common application is mapping a gene’s function by altering its gene expression and assaying cellular phenotypic change under some applied selective condition (Figure 1A). CRISPRi is particularly useful for investigating essential genes because its repression can be titrated to prevent full knockdown and cell death (Knoot et al., 2020; Bosch et al., 2021). Additionally, epistatic effects of multiple genes can easily be investigation by simply expressing multiple gRNA within the same cell (Ellis et al., 2021; McNeil et al., 2022). Although not as common as CRISPRi due to stricter design rules (Fontana et al., 2020a), CRISPRa can be used to induce expression of silent genes to investigate their functions and products, including entire silent biosynthetic gene clusters (Ke et al., 2021). Combined, these are the most common use of CRISPRi/a tools in non-model bacteria, with 80 reports across six phyla (Table 1) (Behler et al., 2018; Stamsås et al., 2018; Ke et al., 2021). The recent development of Mobile-CRISPRi (Peters et al., 2019), CRAGE-CRISPR (Ke et al., 2021), and a workflow for introducing genetic manipulation tools into non-model gut bacteria (Jin et al., 2022) will facilitate the expansion of CRISPRi/a tools into new species and strains, including recalcitrate pathogens and novel species without sequenced genomes.

Additionally, CRISPRi/a can be used to control transcription regulatory networks, such as genetic circuits, by designing and expressing gRNA to regulate the output promoter for each logic gate or node (Figure 1B). CRISPRi/a is especially effective for controlling complex synthetic transcription regulatory networks as the gRNA can be designed to target nearly any arbitrary sequence with an appropriate PAM (or equivalent) sequence (Taketani et al., 2020; Ellis et al., 2021). CRISPRi/a circuits can be fully synthetic and auxiliary to the native genetic regulatory networks, such as a heterologous sensor or multi-input circuit that senses and responds to external inputs in complex environments (Mimee et al., 2015; Taketani et al., 2020). Alternatively, CRISPRi/a can be interfaced with native gene regulatory systems to control the host’s metabolism in response to external stimuli, such as cell density, through either heterologous (Liu Y. et al., 2020) or even indigenous sensor systems (Tian et al., 2020). However, caution must be taken to prevent the expression of too many gRNA at once since they compete over the limited dCas protein resource and, thus, can decrease the repression of target genes (Del Vecchio et al., 2008; Li et al., 2018; Zhang and Voigt, 2018). Synthetic CRISPRi/a regulatory networks are rare in non-model bacteria, having been reported in only seven studies across four phyla, and primarily incorporate CRISPRi (Table 1). However, a single CRISPRa genetic circuit in *Salmonella* has been reported (Bhokisham et al., 2020).

CRISPRi/a tools have also been used to redirect carbon and energy flow for metabolic engineering in non-model bacteria (Figure 1C). CRISPRi is often used to repress a native gene(s), including essential genes, to redirect carbon flux towards a desired product (Wang et al., 2017; Shabestary et al., 2018) or bioactive molecule (Yu et al., 2018; Liu et al., 2021b). CRISPRa can be used to activate the desired metabolic pathway to increase biosynthesis of the desired product, such as an anti-cancer drug in a weakly-expressed biosynthetic gene cluster (Peng et al., 2018; Ye et al., 2019). In most examples, the CRISPRi/a components are constitutively expressed, yet some studies employ dynamic metabolic engineering strategies by utilizing inducible systems and/or genetically encoded biosensors to switch between cell growth and product biosynthesis states to improve production (Liu Y. et al., 2020; Tian et al., 2020; Shabestary et al., 2021). These tools can be used to tune endogenous metabolism and/or heterologous metabolic pathways (Peng et al., 2018; Banerjee et al., 2020). CRISPRi/a tools are most often combined with other metabolic engineering techniques, such as the deletion, overexpression, or mutation of select genes and optimization of medium, to further increase titers of the desired product (Park et al., 2019; Dietsch et al., 2021; Kozaeva et al., 2021).

Large-scale CRISPRi screens and selections have been developed to investigate genotype-phenotype relationships through gRNA fitness (Figure 1D). These assays can use small, targeted libraries, such as essential genes or genes in a metabolic pathway (Shields et al., 2020; Göttl et al., 2021), or large genome-wide libraries targeting nearly all genes in the bacterial genome (Lee et al., 2019; Jiang et al., 2020). Additionally, CRISPRi libraries can be constructed in two major forms—pooled libraries, where cells containing different gRNA are mixed during library construction (Bosch et al., 2021; Rahman et al., 2021), a strategy known as multiplexing, or arrayed libraries where different gRNA designs are constructed individually in different clonal populations, typically arrayed in microtiter plates (Liu et al., 2017; Göttl et al., 2021). Pooled competitive selections are more common due to the ease of DNA construction and analysis of large, genome-scale gRNA libraries with >10,000 designs by next-generation sequencing (Lee et al., 2019; Bosch et al., 2021). However, because all cells directly compete in pooled competitive growth assays, “cheaters” may arise that take advantage of different strain interactions, so the results of any individual gRNA design should be verified in isolation (Yao et al., 2020; Liu X. et al., 2021). Additionally, the results from these pooled CRISPRi screens or selections are specific to the gRNA design and not the target gene since confounding effects (i.e., off-target effects) could produce false positives or negatives, so careful design of gRNA libraries is vital (Cui et al., 2018; Wang T. et al., 2018). Genome-wide CRISPRi screens or selections are relatively uncommon (Table 1). While not demonstrated to date, genome-wide bacterial CRISPRa is theoretically possible, provided the design rules for activation are met (Fontana et al., 2020a).

## Conclusion and Perspectives

CRISPRi has been established in non-model bacteria across eight phyla and applied from small, single gene functional studies to large genome-wide screens. The creation of new tools and protocols for introducing CRISPRi/a into non-model bacteria will facilitate the continuation of this rapid expansion. Several novel and exciting CRISPRa tools with greater activation and unique characteristics have been developed recently in both model and non-model bacteria, yet there remains a need for stronger and more versatile bacterial CRISPRa tools, especially for the activation of native genes. These bacterial CRISPRa tools have lagged behind the development of both eukaryotic CRISPRa tools and bacterial CRISPRi tools. However, the recent development of several new CRISPRa systems with less stringent design rules and higher levels of activation (>10-fold) shows great promise for effective, tailored gene activation in bacteria (Liu Y. et al., 2019; Fontana et al., 2020a; Ho et al., 2020; Villegas Kcam et al., 2021). These CRISPRa technologies were created using directed evolution and thorough tool design. Further improvements could be achieved by creating CRISPRa tools from CRISPR systems with more relaxed PAM requirements, directed evolution of CRISPRa components (activator domain, gRNA scaffold(s), and dCas protein) for greater activation, and high-throughput screening of gRNAs and promoters to uncover additional nuanced design rules for a given tool. CRISPRa has the potential to become a more effective and widely used tool for programmable gene activation in both model and non-model bacteria for a variety of industrial and research applications, such as metabolic engineering and elucidation of gene function. While many CRISPRi/a approaches in non-model bacteria have been established using genetic parts that are not well-defined or characterized, the creation of comprehensive genetic part toolboxes for these strains, which are vital for the rational design and precise control of CRISPRi/a tools, will accelerate further development and optimization of the tools. Finally, CRISPRi/a approaches have primarily been developed for more genetically tractable strains of non-model bacteria. There is a need for efficient workflows to domesticate and introduce CRISPRi/a tools to novel bacterial species and strains. Despite these current challenges, CRISPRi/a technology remains a versatile approach for programmable transcriptional regulation in non-model bacteria.
